# Cholesteryl ester transfer protein (CETP) I405V polymorphism and cardiovascular disease in eastern European Caucasians – a cross-sectional study

**DOI:** 10.1186/s12877-016-0318-y

**Published:** 2016-07-20

**Authors:** Jasmin Bustami, Anna Sukiasyan, Juozas Kupcinskas, Jurgita Skieceviciene, Leonid Iakoubov, Malgorzata Szwed, Christoph Kleinle, Ralf R. Schumann, Monika Puzianowska-Kuznicka, Lutz Hamann

**Affiliations:** Institute of Microbiology and Hygiene, Charité University Medical Center Berlin, Rahel-Hirsch-Weg 3, 10117 Berlin, Germany; Research Institute of Epidemiology, MOH, Yerevan, Armenia; Department of Gastro-enterology, Lithuanian University of Health Sciences, Kaunas, Lithuania; Institute for Digestive Research, Lithuanian University of Health Sciences, Kaunas, Lithuania; Cellecta Inc, Mountain View, California, USA; Department of Human Epigenetics, Mossakowski Medical Research Centre, Polish Academy of Sciences, Warsaw, Poland; Department of Geriatrics and Gerontology, Medical Centre of Postgraduate Education, Warsaw, Poland

**Keywords:** Cholesteryl ester transfer protein, Genetic polymorphism, High-density lipoprotein, Single nucleotide polymorphism, Cardio vascular disease

## Abstract

**Background:**

The cholesteryl ester transfer protein (CETP) polymorphism I405V has been suggested to be involved in longevity and susceptibility to cardiovascular diseases. An enhanced reverse cholesterol transport due to enhanced HDL levels has been hypothesized to be the underlying mechanism. However, clinical trials with HDL-enhancing drugs failed to show beneficial effects. Consequently, it has been postulated that genetic variations enhancing HDL levels are cardioprotective only if they also decrease LDL levels.

**Methods:**

A cross-sectional study was conducted to genotype 1028 healthy blood donors and 1517 clinically well characterized elderly for CETP I405V.

**Results:**

We could not find any association of this polymorphism with age for both, males or females, in any of these cohorts (*P* = 0.71 and *P* = 0.57, respectively, for males and *P* = 0.55 and *P* = 0.88, respectively, for females). In addition, no association with cardiovascular diseases could be observed in the elderly cohort (males OR = 1.12 and females OR = 0.88). In the same cohort, the CETP V405V genotype was associated with significantly enhanced HDL levels (*P* = 0.03), mostly owing to the female sex (*P* = 0.46 for males, *P* = 0.02 for females), whereas LDL and triglyceride levels were unchanged (*P* = 0.62 and *P* = 0.18, respectively).

**Conclusion:**

Our data support the recent hypothesis that variations enhancing HDL levels without affecting LDL levels are not associated with the risk for cardiovascular diseases.

## Background

The process of aging and longevity are complex and far from being understood. Besides lifestyle and environmental factors, numerous genetic factors have been proposed to influence these processes. Several studies showed that healthy aging and particularly longevity, known as aging up to 90 years or more, are highly heritable. For example, siblings of centenarians also have an enhanced probability for a longer healthy life [1, 2]. In addition to variations of mitochondrial and nuclear DNA, also the composition of gut microbiome has been suggested to play an important role for longevity [3]. Variations of nuclear DNA that have been postulated to be associated with aging phenotypes include polymorphisms of genes encoding proteins of the immune system [4], the DNA repair system, particularly variations affecting telomere length [5, 6], metabolism [7, 8], and variations associated with the onset of age-related diseases such as atherosclerosis resulting in cardiovascular diseases (CVD), diabetes mellitus, Alzheimer Disease, and cancer [9].

Among age-associated diseases, CVD plays a pivotal role regarding mortality in the middle-aged population of the Western world [10, 11]. Next to lifestyle, the most accepted risk factor for atherosclerosis is dyslipidemia and high cholesterol levels (reviewed by Steinberg [12–15]). Plasma cholesterol is bound to lipoproteins such as low density (LDL) and high density (HDL) lipoproteins. LDL transports cholesterol into cells, whereas HDL-bound cholesterol is transported to the liver for excretion [16]. The correlation of high cholesterol levels and the susceptibility to CVD led to the development and wide use of cholesterol-lowering drugs, among them the statins, which result in a decreased LDL level [15]. On the other hand, high HDL levels have been found to be protective against CVD [17], supporting the “Reverse Cholesterol Transport” (RCT) concept, according to which HDL-mediated cholesterol transport to the liver results in its fecal excretion and, thereby, in the reduction of plasma cholesterol level [18].

Notably, susceptibility for atherosclerosis shows a strong genetic basis [19, 20]. Several genome-wide association studies have been conducted identifying many risk variants for this condition. As expected, some of these variants affect the lipid metabolism (reviewed by Prins et al. [21]). A unique “lipoprotein genotype”, defined by variations affecting the lipid metabolism, including several polymorphisms in lipoprotein transfer genes, has been suggested to be associated with longevity [22]. Among them, the apolipoprotein E polymorphisms rs429358 and rs7412 determining the three major alleles E2, E3, and E4 of apoE [23, 24], polymorphisms in cholesteryl ester transfer protein (CETP, rs5882) and apolipoprotein CIII (rs2542052) have been shown to influence lipid metabolism and to be associated with age [22, 25, 26].

CETP is a plasma protein that catalyzes the transfer of cholesteryl ester from HDL to other lipoproteins. A point mutation resulting in a splicing defect of CETP pre-mRNA causes a loss of plasma CETP. Subjects homozygous for this mutation exhibit significantly elevated HDL levels [27]. In line with the “Reverse Cholesterol Transport (RCT)” concept, CETP inhibition was hypothesized to be a strategy to increase HDL levels and thereby lowering plasma cholesterol levels and reducing susceptibility for atherosclerosis [28, 29]. Therefore, several CETP inhibitors have been tested in clinical trials. However, a recent meta-analysis revealed that elevation of HDL levels failed to induce a significant benefit regarding protection from atherosclerosis. It was suggested then that elevation of HDL levels alone does not result in an increased RCT rate [30]. Another hypothetical explanation for this failure is that HDL levels are rather a marker or a consequence of CVD without being causally involved in CVD pathogenesis [31].

The CETP SNP rs5882 (I405V) V/V variant has been shown to reduce CETP levels and, according to the RCT hypothesis, should be cardio-protective and, consequently, associated with longevity [32]. However, conflicting results have been published showing the V/V genotype to be either positively associated with longevity in a Jewish population or negatively associated in an Asian population [25, 26, 33]. Apart from longevity, which accounts only for a small part of aging phenotypes, healthy aging up to 70–90 years is another important parameter, since the human population rapidly ages. We here investigated the association of the CETP rs5882 (I405V) SNP with healthy aging and the susceptibility to cardiovascular disease in two study groups, the first group consisting of healthy blood donors from Lithuania and a second study group from Poland consisted of 1517 elderly subjects.

## Methods

### Study subjects

#### Lithuanian cohort

Blood of 1028 healthy voluntary unrelated blood donors was obtained during the years 2008–2012 at the National Blood Center (Kaunas, Lithuania). All participants were of Caucasian ethnicity and were free of chronic disease or permanent medication use. This cohort has been instrumental previously for hemochromatosis gene *HFE* studies [34]. Genotyping was successful in 989 samples.

#### Polish cohort

PolSenior was a multicenter, interdisciplinary project, designed to assess health and socio-economic status of the Polish Caucasians aged ≥65 years. In short, study participants, recruited using three-stage stratified proportional draw, were split into equally-sized age groups (65–69, 70–74, 75–79, 80–84, 85–89 and ≥90 years). Details of the PolSenior recruitment are described elsewhere [35]. Project participants completed a detailed questionnaire regarding their medical, social, and economic past and current status, underwent an examination including elements of comprehensive geriatric assessment, and donated blood for biochemical and genetic analyses. A sub-group of 1517 participants of the PolSenior program, the first ones for whom the complete medical records (including, among others, data on cardiovascular and respiratory diseases, cancer, diabetes, stroke and cognitive impairment) and DNA samples were available at the beginning of current study, was analyzed.

### Genotyping

Genomic DNA was prepared by standard salting-out procedures. The genetic analysis of the CETP rs5882 (I405V) was performed by PCR employing the LightCyler 480™ (Roche Diagnostics) and subsequent melting curve analysis. The PCR reaction contained fluorescence-labeled hybridization FRET probes. Primer and probes were as follows: f-primer: ctccagggaggactcacca, r-primer: cccctccagcccacactta, anchor probe: LC640-cctgcagtcaatg-atcaccgctgt, sensor probe: tccgagtccatccagagct-FL resulting in melting points of 54 °C and 61 °C for the wild-type (V), and the mutated allele (I), respectively.

### Statistics

*P*-values were determined by Chi^2^ test, in case of linear or logistic regression *P*-values were determined by *t*-test or Wald statistic, respectively. Linear and binary logistic regression analyses have been performed by employing the IBM SPSS Statistics software package (version 20.0, IBM, Munich, Germany). The association of CETP genotypes with age within the Lithuanian cohort was done by linear regression with age as dependent variable (normality of the data was assessed using a normality test) and genotypes as independent variable. The association of CETP genotypes with cardio vascular disease was analyzed by logistic regression with cardiovascular disease as dependent variable and genotypes and age as independent variables. Association of CETP genotypes with lipid levels were done age adjusted with lipid levels as outcome variables and genotype as independent variable. Age and lipid levels showed a linear association (data not shown), however, there was no change of lipid levels with age. HDL and LDL levels were normally distributed, only triglyceride levels showed a slight deviation from normal distribution as determined by normality test.

## Results

### No association of CETP rs5882 with age

Baseline characteristic of genotyped subjects are shown in Table 1. Results of genotyping for CETP rs5882 (I405V) indicate that an equal distribution of the genotypes comparing younger (age < 50, mean age: 37.4, SD: 7.3) and older study subjects (age ≥ 50, mean age: 58.4, SD: 7.7, Table 2) cannot be ruled out. The cut-off was set at 50 years because the incidence of age-related diseases significantly increases after this age. Since sex is the most accepted factor associated with longevity, with females reaching a significantly higher mean age than males, we stratified our groups for sex. After this stratification we also failed to find any significant association with age by linear regression analysis, by comparing the V/V genotype versus I/I + I/V genotypes for both, males (*P* = 0.71) and females (*P* = 0.55).

**Table 1:** Baseline characteristics of study subjects

	All	Age < 50 *N* = 736	Age ≥ 50 *N* = 253
Lithuanian cohort (*N* = 989)			
Age range Mean age (SD)	25–84 42.8 (11.8)	25–49 37.4 (7.3)	50–84 58.4 (7.7)
Lower quartile/median/upper quartile	34/42/50	31/38/44	52/56/64
Male/Female	609/380	494/242	115/138
PolSenior group (*N* = 1517)			
Age range Mean age (SD)	65–92 76.77 (6.4)		
Males/Females	796/721		
Disease free (%)	508 (33.5)		
Cardiovascular disease (%)	485 (32.0)		
Cancer (%)	90 (5.9)		
Lung disease (%)	249 (16.4)		
MMSE < 24 (%)	399 (26.3)		
DM (%)	121 (8.0)		

*MMSE* Polish version of the mini-mental state examination test, *DM* diabetes mellitus

**Table 2:** CETP rs5882 (I405V) genotype distribution in the study cohorts

	Younger subjects (Age < 50)			Older subjects (Age ≥ 50)			*P*-value
	I/I (%)	V/I (%)	V/V (%)	I/I (%)	V/I (%)	V/V (%)	
Lithuanian cohort	354 (48.1)	304 (41.3)	78 (10.6)	141 (55.7)	89 (35.2)	23 (9.1)	0.11
PolSenior group				803 (52.0)	601 (38.9)	113 (7.3)	

The *P*-value for comparison of the three genotypes was determined by Chi^2^

In addition, we analyzed the group composed of 1517 Polish Caucasians, covering age 65 to 92, for an association of the CETP V/V genotype with age. Also in this cohort, we failed to detect any association of this SNP with age in both males (*P* = 0.57) and females (*P* = 0.88) (Table 3).

**Table 3:** No associations of the CETP rs5882 V/V genotype with age

	Male			Female		
	*P*-value	B	95 % CI	*P*-value	B	95 % CI
Lithuanian cohort						
V/V versus I/I + I/V	0.71	−0.02	−1.63–1.11	0.55	−0.03	−2.80–1.49
PolSenior group						
V/V versus I/I + I/V	0.57	−0.02	−1.06–0.59	0.88	−0.15	−0.99–0.85

Association of age with genotypes was performed by univariate linear regression comparing the less functional homozygote genotype with the others (recessive model). The I/I + I/V genotypes were used as reference

### No association of CETP rs5882 with cardiovascular disease

In order to analyze the association of the CETP rs5882 (I405V) SNP with the occurrence of cardiovascular diseases, we analyzed the 1517 PolSenior study participants, for whom detailed medical reports were available. Comparison of the genotypes between subjects with cardiovascular diseases (mean age: 76.8 years) and disease-free subjects (mean age: 76.7years) revealed only marginal differences in the genotype distribution with V/V in subjects with CVD and disease free subjects of 7.2 % and 7.6 %, respectively. By logistic regression comparing the I/I + I/V versus V/V genotypes for males and females we failed to reveal any significant associations (males: OR: 1.12, females: OR: 0.80). The co-variable age is also not significantly associated with cardiovascular disease (males: OR: 1.01, females: OR: 1.00) (Table 4).

**Table 4:** No association of the CETP rs5882 V/V genotype with cardiovascular disease within the PolSenior group

	OR	CI (95 %)
Female		
CETP 405 V/V versus I/I + I/V	0.80	0.40–1.60
Age	1.00	0.96–1.02
Male		
CETP 405 V/V versus I/I + I/V	1.12	0.62–2.02
Age	1.01	0.99–1.04

Odds ratios (OR) for cardiovascular disease were determined by logistic regression with disease status as dependent variable and the genotypes as independent variable. The combined I/I and I/V genotypes were used as reference, and the V/V genotype as predictor. Age was included as co-variable

### Association of lipid levels with CETP rs5882 genotypes in the PolSenior group

Since it has been postulated that CETP rs5882 (I405V) genotypes influence HDL levels thereby potentially influencing the risk of cardiovascular diseases [32], we analyzed lipid levels according to CETP genotypes in the PolSenior group. Subjects with cardiovascular diseases were excluded due to lipid lowering therapy. As expected, the CETP V/V genotype resulted in an age-adjusted significantly enhanced HDL level (*P* = 0.03). This increased HDL level was more pronounced and significant in females (*P* = 0.02), and noticeable but not significant in males (*P* = 0.46). No significant changes in LDL and triglyceride levels could be observed (*P* = 0.62 and *P* = 0.18, respectively, Table 5). The low r^2^ values for all associations indicate that genotypes as well as age as co-variable has low impact on lipid levels. Analyzing lipid levels in different age groups (65–74 years, 75–84 years, and 85–95 years) by Kruskal Wallis test also showed no significant association (data not shown).

**Table 5:** Association of the CETP rs5882 V/V genotype with lipid levels

All	I/I + I/V (*n* = 937)	V/V (*n* = 77)	*P*-value	r^2^
HDL mean mg/dl (SD)	50.3 (13.2)	53.8 (17.0)	**0.03**	0.007
LDL mean mg/dl (SD)	126.4 (38.3)	128.9 (42.6)	0.62	0.005
Triglycerides mean mg/dl (SD)	128.4 (58.1)	138.5 (91.4)	0.18	0.010
Female	I/I + I/V (*n* = 464)	V/V (*n* = 39)	*P*-value	r^2^
HDL mean mg/dl (SD)	51.7 (12.9)	56.9 (16.5)	**0.02**	0.011
LDL mean mg/dl (SD)	129.2 (40.8)	132.8 (44.5)	0.94	0.001
Triglycerides mean mg/dl (SD)	138.7 (60.6)	145.1 (86.2)	0.56	0.007
Male	I/I + I/V (*n* = 473)	V/V (*n* = 38)	*P*-value	r^2^
HDL mean mg/dl (SD)	48.8 (13.3)	50.7 (17.3)	0.46	0.009
LDL mean mg/dl (SD)	123.6 (35.6)	124.9 (40.6)	0.92	0.024
Triglycerides mean mg/dl (SD)	118.3 (40.8)	131.9 (97.2)	0.19	0.018

Age-adjusted *P*-values were determined by linear regression. Subjects with cardiovascular diseases were excluded due to lipid lowering therapy. *r* ^*2*^ = coefficient of determination, SD = standard deviation, significant values are shown bold

## Discussion

An association of the CETP rs5882 (I405V) SNP with longevity and aging-related morbidity has been published with different results, suggesting the V/V genotype to be protective in one study, or a risk factor in others for example for CVD [25, 26, 33]. The hypothesized mechanism is that elevated HDL levels found in subjects with the V/V genotype may protect from CVD and therefore result in a positive association of this genotype with healthy aging and longevity [22]. Here we investigated a cohort of middle aged healthy subjects and a population-based cohort of aged subjects exhibiting the typical distribution of age-related diseases, for an association of CETP I405V with age and CVD. Although we found a significantly enhanced HDL level in CETP 405 V/V carriers, we failed to find any association of this genotype with age or CVD for both males and females. A limitation of our study is the limited sample size, therefore, we cannot rule out any effect of the CETP I405V genotype on CVD or an association with age.

One potential explanation of this discrepancy between our and some of the previously published studies may be the different genetic background of the study populations. On the other hand, however, our negative results may also reflect recent findings suggesting that HDL levels may be a risk marker, but not a causal risk factor for CVD [36]. Several other variants in genes affecting lipid metabolism associated with elevated HDL levels failed to display any association with CVD, with the exception of those SNPs that were also associated with decreased LDL or triglycerides levels. One example is the CETP promoter SNP rs3764261. This SNP results, in addition to elevated HDL levels, in decreased LDL and triglyceride levels and is, therefore, associated with a decreased risk for CVD [31]. Our results showed that the CETP rs5882 (I405V) was associated with a significantly elevated HDL level. LDL or triglyceride levels, however, were unchanged. Therefore, the lack of any association with CVD in our study is in agreement with the results of other studies analyzing other HDL-elevating SNPs that do not change LDL or triglyceride levels [31]. In line with this, two recent trials testing the HDL-elevating drugs niacin and dalceptrapib were terminated prematurely because no clinical benefit could be observed in subjects with established CVD [36]. Even the reported association of CETP rs5882 (I405V) with healthy aging or longevity should be taken with care, particularly since the hypothesized mechanism of protection from CVD could not be confirmed. However, our study is too small and needs larger confirmation studies to exclude any association of CETP rs5882 (I405V) with healthy aging and, furthermore, other than the described above mechanisms could be responsible for such an association.

## Conclusion

In our cohort we could not find evidence for an association of CETP rs5882 (I405V) with CVD or LDL levels. This is in line with the current hypothesis that only SNPs affecting LDL levels or both, LDL and HDL levels, protect from CVD. In line, we also failed to find an association of the HDL-increasing genotype of CETP rs5882 (I405V) with aging not complicated by CVD.

## Abbreviations

CETP, cholesteryl ester transfer protein; CVD, cardio vascular disease; FRET, fluorescence resonance energy transfer; HDL, high density lipoprotein; LDL, low density lipoprotein; RCT, reverse cholesterol transfer
